# Epidemiology and Survival of Esophageal Cancer Patients in an American Cohort

**DOI:** 10.7759/cureus.2507

**Published:** 2018-04-19

**Authors:** Ammar Nassri, Hong Zhu, Mayssan Muftah, Zeeshan Ramzan

**Affiliations:** 1 Department of Medicine Gastroenterology, UF Jacksonville; 2 Department of Clinical Science, Simmons Comprehensive Cancer Center, University of Texas Southwestern Medical Center, Dallas, TX; 3 Department of Internal Medicine, Emory University School of Medicine; 4 Gastroenterology, Department of Medicine, University of Texas Southwestern Medical Center, Dallas, TX

**Keywords:** esophageal cancer, oncology, surgery, ivor lewis esophagectomy, prognosis, epidemiology

## Abstract

Objectives

This study seeks to delineate trends in esophageal cancer patients in an American cohort and, in particular, examine the impact of race and histology on survival.

Methods

The association between over 50 variables between histology and race subgroups was evaluated. Survival was calculated using Kaplan-Meier curves and a multivariable Cox regression analysis (MVA) was performed.

Results

Poorer survival was noted in black vs. white (193 ± 65 days vs. 254 ± 39, 95% CI 205-295, p=0.07) and squamous cell cancer (SCC) vs. adenocarcinoma (AC) (233 ± 24 days vs. 303 ± 48, 95% CI 197-339, p=0.01) patients. In patients with resectable cancer, blacks had poorer survival than whites (253 ± 46 days vs. 538 ± 202, 95% CI 269-603, p=0.03), and SCC had poorer survival than AC (333 ± 58 vs. 638 ± 152 days, 95% CI 306-634, p=0.006). A higher percentage of white patients received surgery compared to black patients (36% vs. 8%, p=0.08). MVA revealed that only surgery was an independent predictor of mortality (p=0.001).

Conclusion

Black race and SCC were associated with poorer survival. On MVA, surgery was an independent predictor of mortality. Clinicians should be aggressive in offering potentially curative procedures to patients and eliminating socioeconomic barriers.

## Introduction

Esophageal cancer (EC) is one of the most common cancers diagnosed, with an estimated global incidence of 455,800 [1]. In the United States, it is estimated that there will be 17,290 new cases and 15,850 deaths in 2018 [2]. In addition to the rise in incidence over the past few decades [3], there appears to be a concurrent change in histological type, with studies reporting a 463% increase in the incidence of adenocarcinoma (AC) in Americans in 2000-2004 as compared to 1975-1979 [4]. Adenocarcinoma has replaced squamous cell carcinoma (SCC) as the most common type of esophageal carcinoma, in part due to an increase in risk factors for AC such as obesity and gastroesophageal reflux disease (GERD) [5]. The tumor biology of EC is still not fully understood, and patients with SCC have been shown to have significantly poorer survival than patients with AC [6]. Efforts at elucidating the underlying mechanisms as well as developing biomarkers for early detection and screening have proved daunting [7]. Moreover, there seems to be a racial disparity in survival between white and black patients with esophageal carcinoma for reasons that are still not fully clear, with the age-adjusted incidence and death for blacks almost twice that for whites [8].

This study uniquely analyzes symptoms and risk factors at presentation as well as various demographic, pathological, clinical, and laboratory variables. In addition to delineating trends in the presentation and treatment of esophageal cancer patients in an American cohort, it seeks to closely examine the impact of race and histology on survival in our veteran patient population.

## Materials and methods

The variables assessed in the study are shown in Table 1.

**Table 1:** Variables Included in the Study EMR: endoscopic mucosal resection; BMI: body mass index; WBC: white blood cell; ECOG: Eastern Cooperative Oncology Group

Demographic Variables	Laboratory Values	Clinical Variables	Symptoms at Presentation	Risk Factors	Treatment
Age	Albumin at Diagnosis	Survival Days	Hoarseness	History of Alcohol Use	Surgery
Race	WBC at Diagnosis	Age at Diagnosis	Fatigue	History of Tobacco Use	EMR
Gender		Stage at Diagnosis	Regurgitation	History of GERD Symptoms	Postop Adjuvant Therapy
BMI at Diagnosis		ECOG Functional Status	Weight loss	History of H pylori	Neoadjuvant Therapy
		Histological Type	Chest pain	On PPI at time of Diagnosis	Definitive Chemoradiation
		Anatomical Location of Cancer	Dysphagia to solids	History of Cholecystectomy	Stent Placement
			Dysphagia to solids and liquids	Prior Gastrectomy	Gastric/Jejunal Tube
			Heart Burn	History of Atrophic gastritis	Palliative Therapy
			Nausea/Vomiting	History of Head/Neck Cancer	Any Chemoradiation
			Hematemesis	Family History	
			Hematochezia/Melena	History of Achalasia	
			Anemia	History of Esophagogastric Cancer	
			Abdominal Pain		
			Odynophagia		
			Neck mass		
			Abdominal mass		
			Neurological symptoms		

Survival was calculated based on the date of pathological diagnosis obtained from pathology records and the date of demise and, where unavailable, a cutoff date of September 01, 2013 was used. Presenting complaints and risk factors were abstracted from the medical provider notes on the patient’s computerized charts. Laboratory values and demographic values were obtained on the date closest to pathological diagnosis durin­g that hospital admission. Eastern Cooperative Oncology Group (ECOG) scores and treatment regimen were abstracted from the oncology notes and the location of cancer from the endoscopy notes. The location of the cancer was obtained from the endoscopy notes, and a value of 30 cm from the incisors was used as the demarcation point between upper and lower tumors. The association between each clinical, demographic, and laboratory factor between subgroups was evaluated by Fisher’s exact test or the chi-square test for categorical variables, and by the two sample t-tests for continuous variables. Survival was calculated using Kaplan-Meier curves, and survival between groups was compared using the log-rank test. All analyses were performed using SPSS (IBM, Armonk, New York, United States). Statistical significance was determined at p ≤ .05. A p-value of <0.1 was considered marginally significant. This study was approved by the Institutional Review Board (IRB) at our institution.

## Results

Demographics and epidemiology

A total of 122 patients with esophageal cancer were identified. Their features are presented in Table 2. 

**Table 2:** Demographics BMI: body mass index; ECOG: Eastern Cooperative Oncology Group; EMR: endoscopic mucosal resection; GERD: gastroesophageal reflux disease; PPI: proton pump inhibitor; SEM: standard error of the mean; WBC: white blood cell

	n (SEM/%)		n (SEM/%)
Mean Age at Diagnosis (years)	64.0 (0.9)	Interventions	
Mean Age at Demise (years)	65.1 (1.0)	Surgery	19 (16%)
Sex		EMR	7 (6%)
Male	119 (97.5%)	Postop Adjuvant Therapy	2 (2%)
Female	3 (2.5%)	Neoadjuvant Therapy	25 (20.5%)
Race		Definitive Chemoradiation	25 (20.5%)
White	92 (75.4%)	Stent Placement	26 (21.3%)
Black	25 (20.5%)	Gastric/Jejunal Tube	51 (41.8%)
Hispanic	4 (3.3%)	Palliative therapy	39 (32%)
Unknown	1 (0.8%)	Risk Factors	
Mean Weight Diagnosis (lbs)	178.3 (4.6)	Alcohol	90 (73.8%)
Mean BMI Diagnosis	26.0 (0.7)	Tobacco	101 (82.8%)
Mean Albumin at Diagnosis (g/dl)	3.6 (0.06)	GERD	53 (43.4%)
Mean WBC at Diagnosis (K/µL)	8.7 (0.3)	H pylori	5 (4.1%)
Histology		On PPI	35 (28.7%)
Adenocarcinoma	75 (61.5%)	Cholecystectomy	11 (9.0%)
Squamous Cell Carcinoma	41 (33.6%)	Prior gastrectomy	0
Undifferentiated	6 (4.9%)	Atrophic gastritis	0
Location		Head/Neck cancer	4 (3.3%)
Upper	32 (26.2%)	Family history Esophageal cancer	7 (5.7%)
Lower	90 (73.8%)	Achalasia	3 (2.5%)
Stage at Diagnosis		Prior Esophageal/Gastric cancer	4 (3.3%)
I	18 (14.8%)	Symptoms at Presentation	
II	19 (15.6%)	Hoarseness	8 (6.6%)
III	26 (21.3%)	Fatigue	17 (13.9%)
IV	50 (41.0%)	Regurgitation	22 (18%)
Unknown	9 (7.4%)	Weight loss	68 (55.7%)
ECOG at Diagnosis		Chest pain	14 (11.5%)
0	23 (18.9%)	Dysphagia to solids	49 (40.2%)
1	39 (32.0%)	Dysphagia to solids & liquids	37 (30.3%)
2	19 (15.6%)	Heart burn	21 (17.2%)
3	17 (13.9%)	Nausea/Vomiting	12 (9.8%)
4	3 (2.5%)	Hematemesis	8 (6.6%)
Unknown	21 (17.2%)	Hematochezia/Melena	12 (9.8%)
Survival ( days)		Anemia	7 (5.7%)
Mean	591 ±(76 )	Abdominal pain	9 (7.4%)
Median	253± (28 )	Odynophagia	14 (11.5%)
		Neck mass	1 (0.8%)
		Abdominal mass	1 (0.8%)
		Neurological symptoms	1 (0.8%)

The mean age was 64 ± 0.9 years and the mean body mass index (BMI) at diagnosis was 26 ± 0.7. The cohort was predominantly male (97.5%) and white (75.4%). AC was found in 61.5% of the patients and 33.6% had SCC. The majority of the cancers were in the lower esophagus (73.8%) as compared to the upper esophagus (26.2%).

The patients presented mostly with advanced stage carcinoma with almost half (41%) presenting with metastatic disease. The vast majority of patients had a documented history of tobacco (82.8%) and alcohol (73.8%) use. A significant number also had gastroesophageal reflux disease (GERD) (43.4%), whereas only 28.7% were on a proton pump inhibitor. The most common complaint at presentation was weight loss (55.7%) followed by dysphagia to solids (40.2%). In terms of interventions, 41.8% patients received a gastric or jejunal feeding tube, 21.3% received esophageal stents, 15.6% patients had an esophagectomy, and 66.4% patients received some form of chemotherapy or radiation.

Survival

On a Kaplan-Meier analysis, overall median survival for the entire cohort was 253 ± 28 days. 

Histology

When the cohort was stratified by histology, patients with AC had longer median survival than those with SCC (303 ± 48 days vs. 233 ± 24, 95% CI 197-339, p=0.01). In patients with potentially resectable cancer (Stage I-III), SCC patients had significantly poorer survival than AC (333 ± 58 vs. 638 ± 152 days, 95% CI 306-634, p=0.006)

Race

When only black and white patients were compared, whites had a longer median survival (254 ± 39 vs. 193 ± 65 days, 95% CI 205-295, p=0.07). However, when black and whites with potentially resectable cancers (Stage I-III) were compared, there was a statistically significant difference in survival between whites and blacks (538 ± 202 vs. 253 ± 46 days, 95% CI 269-603, p=0.03).

Stage

As expected, there was an inverse relationship between stage of disease at presentation and survival with a median survival of 1447 ± 651 days, 402 ± 137 days, 292 ± 53 days, and 134 ± 35 days for Stage I, II, III, and IV patients, respectively (95% CI 199-307, p<0.0001).

Resectability

Patient survival was stratified by resectability. Stage IV patients were considered to have nonresectable tumors (NR). Patients that had potentially resectable disease (defined as Stage I-III) were grouped into patients that received an esophagectomy (R+SURG), endoscopic mucosal resection (R+EMR), and patients that did not receive surgical/endoscopic intervention (R-). Patients with an unknown stage were not included in the analysis. Patients with NR cancer had poorer survival compared to patients with resectable cancer (stage I-III) (134 ± 45 vs. 470 ± 84 days, 95% CI 193-343, p<0.0001).

There was a clinically and statistically significant difference in survival between the NR (stage IV) group (134 ± 35 days) and resectable (I-III) groups (R-:364 ± 81 days, R+SURG: 733 ± 342 days, R+EMR: 2212 ± 625 days, p<0.0001).

 Among the group that was potentially resectable (stages I-III), the median survival for R- patients compared with patients who received any kind of surgical/endoscopic intervention (R+Surg and R+EMR) was 364 ± 81 days vs. 1044 ± 278 days (95% CI 193-343, p<0.0001).

**Figure 1:**
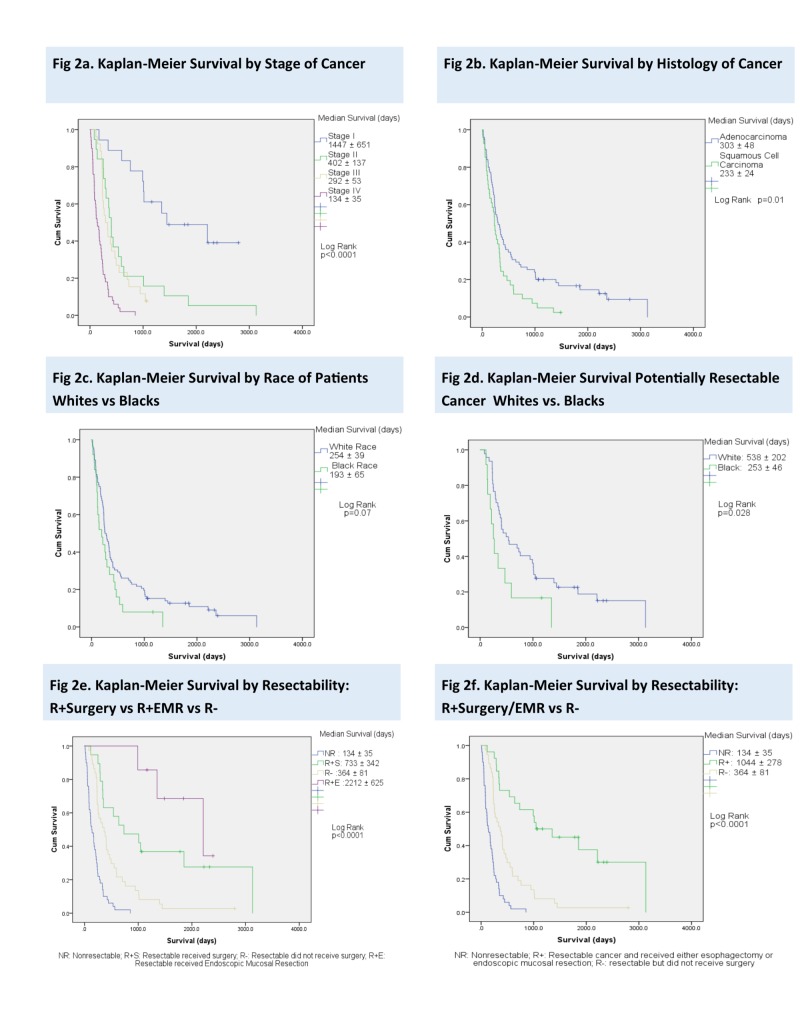
Kaplan-Meier Survival Curves

This data is represented in Figure 1.

Univariate analysis

By Race

A univariate analysis was conducted to elucidate any differences in any of the measured variables between white and black patients (Table 3).

**Table 3:** Univariate Analysis by Race * mean (+SEM); BMI: body mass index; ECOG: Eastern Cooperative Oncology Group; EMR: endoscopic mucosal resection; GERD: gastroesophageal reflux disease; SEM: Standard error of the mean; WBC: white blood cells

	White (n=92)	%	Black (n=25)	%	p-value
Demographics & Clinical Variables					
Adenocarcinoma (AC)	70	76%	3	12%	<0.0001
Squamous Cell Carcinoma (SCC)	18	20%	20	80%	
Upper Esophagus	21	23%	10	40%	0.08
Lower Esophagus	71	77%	15	60%	
Hypoalbuminemia	31	34%	8	32%	0.9
WBC*	9.0+0.4	.	7.5+0.5	.	0.6
Albumin *	3.64+0.07	.	3.46+0.1	.	0.2
Age*	64.4+1.1	.	63.6+2	.	0.7
BMI*	26.42+0.8	.	23.5+1.3	.	0.8
ECOG*	1.38+0.12	.	1.48+0.25	.	0.7
Stage I	13	14%	3	12%	0.6
Stage II	16	17%	2	8%	.
Stage III	18	20%	7	28%	.
Stage IV	39	42%	10	40%	.
Interventions					
Surgery	17	18%	1	4%	0.1
EMR	5	5%	2	8%	0.6
Postop Adjuvant Therapy	1	1%	0	0%	0.8
Neoadjuvant Therapy	20	22%	5	20%	0.9
Definitive Chemoradiation	19	21%	6	24%	0.7
Stent Placement	19	21%	7	28%	0.4
Gastric/Jejunal Tube	40	43%	10	40%	0.8
Palliative therapy	29	32%	9	36%	0.7
Any Chemo/Rad	62	67%	17	68%	1.0
Risk Factors					
Alcohol	69	75%	18	72%	0.8
Tobacco	79	86%	20	80%	0.5
GERD	40	43%	10	40%	0.8
H pylori	4	4%	1	4%	0.7
On Proton Pump Inhibitor	24	26%	8	32%	0.6
Cholecystectomy	8	9%	2	8%	0.6
Prior Gastrectomy	0	0%	0	0%	.
Atrophic Gastritis	0	0%	0	0%	.
Head/Neck Cancer	3	3%	1	4%	0.6
Family History Esophageal Cancer	6	7%	1	4%	0.5
Achalasia	2	2%	1	4%	0.5
Prior Esophageal/Gastric Cancer	2	2%	1	4%	0.5
Symptoms at Presentation					
Hoarseness	7	8%	1	4%	0.5
Fatigue	16	17%	1	4%	0.1
Regurgitation	16	17%	6	24%	0.5
Weight Loss	51	55%	16	64%	0.4
Chest Pain	10	11%	4	16%	0.5
Dysphagia to Solids	32	35%	16	64%	0.008
Dysphagia to Solids and Liquids	32	35%	4	16%	0.1
Heart Burn	17	18%	3	12%	0.5
Nausea/Vomiting	9	10%	2	8%	0.6
Hematemesis	5	5%	2	8%	0.6
Hematochezia/Melena	7	8%	4	16%	0.2
Anemia	6	7%	0	0%	0.3
Abdominal Pain	8	9%	0	0%	0.2
Odynophagia	9	10%	5	20%	0.2
Neck Mass	0	0%	1	4%	0.2
Abdominal Mass	0	0%	1	4%	0.2
Neurological Symptoms	1	1%	0	0%	0.8

Hispanic patients were excluded due to low sample size. There were similar rates of smoking (86% vs. 80%, p=0.47) and alcohol use (75% vs. 72%, p=0.8) between whites and blacks. The majority of white patients presented with AC compared to black patients (76% vs. 12%, p<0.0001), whereas black patients were more likely to present with SCC compared to whites (80% vs. 20%, p<0.0001). Black patients with SCC compared to white patients with SCC had similar rates of alcohol (80% vs. 83%) and tobacco use (80% vs. 100%). There was no significant difference in the stage of presentation at diagnosis between races, with 62% of white patients presenting with advanced stage disease (III+IV) compared to 68% of black patients. A higher percentage of white patients received surgery compared to black patients (18% vs. 4%, p=0.1). Among patients with potentially resectable cancer, whites again received surgery more often than blacks (36% vs. 8%, p=0.08). Out of all variables assessed, only histology (AC: 12% black vs. 76% white, p<0.0001) and dysphagia to solids (64% black vs. 35% white, p=0.008) proved to be statistically significant between whites and blacks.

By Histology

A univariate analysis was conducted to elucidate any differences in any of the measured variables between patients with differing histological subtypes (Table 4).

**Table 4:** Univariate Analysis by Histology * mean (+SEM); BMI: body mass index; ECOG: Eastern Cooperative Oncology Group; EMR: endoscopic mucosal resection; GERD: gastroesophageal reflux disease; SEM: standard error of the mean; WBC: white blood cell

	AC (n=75)	%	SCC (n=41)		%	p value
Demographic & Clinical Variables						
White	70	93%	18	44%		<0.0001
Black	3	4%	20	49%		.
Upper Esophagus	6	8%	24	59%		<0.0001
Lower Esophagus	69	92%	17	41%		.
Hypoalbuminemia	23	31%	16	39%		0.4
WBC *	8.5+0.3	.	8.91+4.6	.		0.6
Albumin *	3.7+0.1	.	3.4+0.1	.		0.3
Age*	64.2+1.2	.	65.0+1.7	.		0.7
BMI*	27.7+ 0.9	.	23.0 + 0.9	.		0.002
ECOG*	1.27 + 0.1	.	1.57+ 0.2	.		0.2
Stage I	13	17%	5	12%		0.2
Stage II	15	20%	4	10%		.
Stage III	13	17%	13	32%		.
Stage IV	31	41%	13	32%		.
Interventions						
Surgery	16	21%	3	7%		0.07
EMR	5	7%	2	5%		0.5
Postop Adjuvant Therapy	2	3%	0	0%		0.5
Neoadjuvant Therapy	15	20%	9	22%		0.8
Definitive Chemoradiation	15	20%	10	24%		0.6
Stent Placement	12	16%	11	27%		0.1
Gastric/Jejunal Tube	30	40%	17	41%		0.9
Palliative Therapy	26	35%	9	22%		0.1
Any Chemo/Rad	52	69%	24	59%		0.2
Risk Factors						
Alcohol	53	71%	33	80%		0.2
Tobacco	62	83%	36	88%		0.5
GERD	35	47%	15	37%		0.3
H pylori	3	4%	2	5%		0.8
On Proton Pump Inhibitor	23	31%	10	24%		0.5
Cholecystectomy	9	12%	1	2%		0.1
Prior Gastrectomy	0	0%	0	0%		.
Atrophic Gastritis	0	0%	0	0%		.
Head/Neck Cancer	2	3%	2	5%		0.6
Family History Esophageal Cancer	6	8%	1	2%		0.4
Achalasia	0	0%	3	7%		0.04
Prior Esophageal/Gastric Cancer	3	4%	1	2%		0.6
Symptoms at Presentation						
Hoarseness	1	1%	6	15%		0.008
Fatigue	15	20%	2	5%		0.03
Regurgitation	14	19%	8	20%		0.9
Weight Loss	38	51%	26	63%		0.2
Chest Pain	8	11%	4	10%		0.6
Dysphagia to Solids	24	32%	22	54%		0.02
Dysphagia to Solids and Liquids	23	31%	13	32%		0.9
Heart Burn	14	19%	5	12%		0.4
Nausea/Vomiting	10	13%	2	5%		0.2
Hematemesis	6	8%	2	5%		0.7
Hematochezia/Melena	7	9%	5	12%		0.6
Anemia	5	7%	2	5%		0.5
Abdominal Pain	8	11%	1	2%		0.2
Odynophagia	6	8%	6	15%		0.3
Neck Mass	0	0%	1	2%		0.4
Abdominal Mass	1	1%	0	0%		0.6
Neurological Symptoms	1	1%	0	0%		0.6

Both smoking (83% vs. 88%, p=0.5) and alcohol use (71% vs. 80%, p=0.2) were equally prevalent in both the AC and SCC groups. There were fewer patients overall with SCC who underwent surgery compared with AC (7% vs. 21%, p=0.07). When looking at patients with potentially resectable cancers, fewer patients in the SCC group received surgery compared with the AC group (14% vs. 45%, p=0.02). There was no significant difference in interventions such as chemotherapy, radiation, enteral tube feeding, or esophageal stenting between AC and SCC patients (see Table 4).

Of the patients in our cohort who had a history of achalasia, all presented had SCC. More patients with SCC complained of dysphagia to solids (p=0.02) and hoarseness (p=0.008) whereas more patients with AC presented complaining of fatigue (p=0.03). Patients with AC had a higher BMI at presentation compared with SCC (27.7+ 0.9 vs. 23.0 + 0.9, p=0.002).

Multivariable analysis

All variables with a univariate analysis p-value < 0.1 obtained on a comparison between the histology and race subgroups were included in a multivariable Cox regression analysis to elucidate predictors of mortality (Table 5).

**Table 5:** Variables Included in the Multivariate Model

Variables	Univariate p value
Race	<0.0001
Surgery	0.07
Histology	<0.0001
Anatomic Location	<0.0001
History of Achalasia	0.04
History of Cholecystectomy	0.1
Hoarseness	0.008
Fatigue	0.03
Dysphagia to Solids and Liquids	0.02

Of the variables included, only surgery (p=0.001) was statistically significant.

## Discussion

Although there was a higher percentage of SCC in blacks as well as a poorer survival rate for SCC compared with AC, the multivariable analysis revealed that histology and race did not play a role in overall survival. The receipt of an esophagectomy was an independent predictor of survival.

Esophagectomy for locally advanced esophageal cancer is currently the standard of care, and neoadjuvant chemoradiation has been shown in several studies to confer a survival advantage [8-10]. Definitive chemoradiation alone has generally not been recommended, although a recent Cochrane meta-analysis suggested that survival outcomes for definitive chemoradiation may be equal to surgery in SCC patients [11]. Several studies have suggested that surgical resection for esophageal cancer is underused [12], likely due to a combination of factors, including limited access to high-volume centers and patient reluctance to undergo surgery.

In our study, on a Kaplan-Meier analysis, there was a statistically and clinically apparent survival benefit of receiving an esophagectomy (p<0.0001). However, in our cohort, there was a total of 52% patients that had potentially resectable tumors (R+ surgery/EMR), but less than half of them (41%) underwent resection. When analyzing causes for not receiving surgery in potentially resectable patients (R-) (Table 6), 16% of the patients were found to have cancer that advanced after neoadjuvant treatment that precluded them from surgery. Almost one-third of the patients (11/38, 29%) refused surgery and were treated solely with chemoradiation despite having a good (ECOG 0-1) functional status. Given the significant survival advantage in patients who received surgery, it seems prudent to be aggressive in offering surgery to patients early and highlighting the survival benefits clearly as well as identifying and eliminating potential socioeconomic barriers.

**Table 6:** Patients with Potentially Resectable Cancer: Reasons for Not Receiving Surgery ECOG: Eastern Cooperative Oncology Group

Reason	n=38	% (/38)
Tumors not amenable to surgery due to local invasion after neoadjuvant	6	16%
Tumor too locally advanced at diagnosis	3	8%
Later found to have metastases and plans for surgery were aborted	3	8%
Lost to follow-up	8	21%
Refused surgery/treated with chemoradiation despite good ECOG	11	29%
Poor performance status	4	11%
Previous gastrointestinal surgery that precluded esophagectomy	3	8%

Although randomized prospective trials are lacking, the use of endoscopic methods instead of surgery for the treatment of early mucosal esophageal cancer have become ubiquitous, with EMR for T1a the most common approach in the United States and increasing in frequency for T1b tumors [13]. Observational studies have demonstrated equal survival rate at one, three, and five years for patients with T1a treated with either endoscopic resection or esophagectomy, although endoscopic therapy is associated with a slightly higher recurrence rate [14]. Similarly, when comparing patients with Stage I cancer who received endoscopic treatment vs. surgery in our study, there was no statistically significant difference in survival noted although this may have been secondary to the limited population size of this subgroup.

Several population-based studies have reported a poorer survival of black patients compared to whites [15-17]. In earlier studies, this was sometimes proposed to be secondary to the increased incidence of SCC (which has poorer survival) in blacks.

However, several epidemiological studies have demonstrated that there may be other factors at play. One study demonstrated worse five-year survival rates for blacks compared to whites (37% vs. 60%, p<0.001). However, on a multivariate analysis and controlling for histology among other factors, the relationship between race and survival was not significant when surgery was taken into consideration [15]. Similarly, a subsequent large analysis of the surveillance, epidemiology, and end results (SEER) database confirmed the disparity in survival and incidence in SCC between black and white patients. However, on a multivariate analysis, the survival disparity disappeared after adjusting for the receipt of esophagectomy [16]. 

Conversely, in a recent study, Taoili et al. confirmed that blacks had a lower rate of esophagectomy; however, the differences in survival persisted even when adjusting for cancer-directed surgery. Interestingly, independent of stage, white and black patients who did not have surgery experienced similar survival, although among patients who did undergo surgery, there was still a poorer overall survival for blacks compared to whites [17]. Possible explanations for this disparity in esophagectomy rates among blacks may be poorer health care delivery, less access to specialized surgeons, poor access to high-volume centers, or secondary to patient refusal. It is also possible that a proportion of these cancers may be less resectable due to the preponderance of SCC in these patients, and thus the location of cancers in the mid-esophagus [15-18].

In our study, there was a significant difference in survival between white and black patients with potentially resectable cancers (Stage I-III), with whites surviving twice as long as blacks (median survival 538 ± 202 vs. 253 ± 46 days, p=0.03, 95% CI 269-603). When comparing overall survival rates between histology groups, we noted differences in survival, with AC patients having superior survival than SCC patients (303 ± 48 days vs. 233 ± 24, 95% CI 197-339, p=0.01). AC has been shown to have superior survival to SCC in the literature [19-20]. In one study that evaluated survival between AC and SCC patients who underwent curative resection, the five-year survival rate was 42.3% vs, 30.3%, p<0.01. This disparity in survival was consistent even in patients who had a complete macroscopic and microscopic resection as well as negative lymph nodes (R0N0) [21]. This poorer survival may be secondary to the higher rate of occult micro-metastases [21], the histology-specific aggressiveness of cancer, or other confounding factors and exposure relating to the patient population, including race.

In our study, almost 80% of blacks presented with SCC compared with 20% of whites (p<0.0001). The higher incidence of SCC in blacks compared to whites has been documented in large population studies [7], but the causes are not fully apparent. Proposed etiologies have involved the lower socioeconomic class (SEC), lower intake of fruits and vegetables, and increased rates of alcohol and tobacco usage [22-23]. However, it is not clear why these factors, while definite risk factors for SCC, result in a higher incidence of SCC in blacks compared with whites. It may be secondary to increased amounts of tobacco and alcohol consumed compared to whites, something hard to accurately quantify in large studies. Furthermore, while lower SEC has been reported to be a risk factor in many studies [22,24-25], it is not exactly clear what the underlying exposures causing these associations are, as low SEC can be considered a surrogate for various lifestyle and environmental exposures, including poor nutrition, poor access to health care, and poor housing [22].

Underlying genetic polymorphisms and race-specific genetic susceptibility may prove to play a significant role in the pathogenesis of SCC. Many recent studies have found various single nucleotide polymorphisms (SNPs) in micro RNA (miRNA) sequences in SCC patients compared with controls [26-27]. These mutations, under the potential effects of factors like alcohol and tobacco, may lead to an alteration of miRNA expression and contribute to carcinogenesis.

Others have reported polymorphic mutations in genes coding for metabolic enzymes, Deoxyribonucleic acid (DNA) repair enzymes and cytokines, which may contribute toward SCC susceptibility [28-30]. While the majority of these studies were done investigating the particularly high incidence of SCC in China and the so-called “esophageal cancer belt,” it may help to explain the increased incidence of SCC in blacks compared to whites.

This study benefits from several strengths. To our knowledge, this is the only cohort of esophageal cancer patients in the United States where such detailed variables, such as potential risk factors and symptoms at presentation, were included, in addition to various laboratory clinical and pathological data across all stages and treatment modalities. In addition, this study’s detailed survival analysis presented in days and stratified by various variables gives providers a better idea of realistic median survival as compared to the five-year survival rates presented elsewhere in the literature. All patients in this cohort were veterans and the VA North Texas Health Care System (VANTHCS) was their primary hospital. All follow-up and treatment were conducted there. This fact, along with the comprehensive and centralized computerized health records, allowed us to collect accurate data endpoints and calculate reliable survival times.

There were a few weaknesses in this study. First, the cohort was of a relatively small sample size and the findings must be interpreted in light of the inherent bias in a retrospective study with a sample of this size. Our population was predominantly male, and of white or black race, and findings cannot be generalizable to other races or women. In addition, it is possible that our patient population had a higher incidence of baseline risks factors (e.g. smoking), which may affect our findings and make them not generalizable to other patient populations.

## Conclusions

In conclusion, there was a significant difference in survival between AC and SCC patients and between White and Black patients with resectable cancer. However, histology and race did not play a role in survival when other factors were controlled in a multivariate analysis. Surgery was found to be an independent predictor of mortality. Given the improved survival benefit of surgery as well as its underuse, clinicians should be more aggressive in offering potentially curative esophagectomies to patients.

## References

[1] Torre LA, Bray F, Siegel RL, Ferlay J, Lortet-Tieulent J, Jemal A (2015). Global cancer statistics, 2012. CA Cancer J Clin.

[2] Siegel RL, Miller KD, Jemal A (2018). Cancer statistics, 2018. CA Cancer J Clin.

[3] Zhang Y (2013). Epidemiology of esophageal cancer. World J Gastroenterol.

[4] Brown LM, Devesa SS, Chow WH (2008). Incidence of adenocarcinoma of the esophagus among white Americans by sex, stage, and age. J Natl Cancer Inst.

[5] Long E, Beales IL (2014). The role of obesity in oesophageal cancer development. Therap Adv Gastroenterol.

[6] Holscher AH, Bollschweiler E, Schneider PM, Siewert JR (1995). Prognosis of early esophageal cancer. Comparison between adeno- and squamous cell carcinoma. Cancer.

[7] Ramzan Z, Nassri AB, Huerta S (2014). The use of imaging and biomarkers in diagnosing Barrett's esophagus and predicting the risk of neoplastic progression. Expert Rev Mol Diagn.

[8] Baquet CR, Commiskey P, Mack K, Meltzer S, Mishra SI (2005). Esophageal cancer epidemiology in blacks and whites: racial and gender disparities in incidence, mortality, survival rates and histology. J Natl Med Assoc.

[9] Morgan MA, Lewis WG, Casbard A (2009). Stage-for-stage comparison of definitive chemoradiotherapy, surgery alone and neoadjuvant chemotherapy for oesophageal carcinoma. Br J Surg.

[10] van Hagen P, Hulshof MC, van Lanschot JJ (2012). Preoperative chemoradiotherapy for esophageal or junctional cancer. N Engl J Med.

[11] Tepper J, Krasna MJ, Niedzwiecki D (2008). Phase III trial of trimodality therapy with cisplatin, fluorouracil, radiotherapy, and surgery compared with surgery alone for esophageal cancer: CALGB 9781. J Clin Oncol.

[12] Best LM, Mughal M, Gurusamy KS (2016). Non-surgical versus surgical treatment for oesophageal cancer. Cochrane Database Syst Rev.

[13] Dubecz A, Sepesi B, Salvador R (2010). Surgical resection for locoregional esophageal cancer is underutilized in the United States. J Am Coll Surg.

[14] Merkow RP, Bilimoria KY, Keswani RN (2014). Treatment trends, risk of lymph node metastasis, and outcomes for localized esophageal cancer. J Natl Cancer Inst.

[15] Wu J, Pan YM, Wang TT, Gao DJ, Hu B (2014). Endotherapy versus surgery for early neoplasia in Barrett's esophagus: a meta-analysis. Gastrointest Endosc.

[16] Greenstein AJ, Litle VR, Swanson SJ, Divino CM, Packer S, McGinn TG, Wisnivesky JP (2008). Racial disparities in esophageal cancer treatment and outcomes. Ann Surg Oncol.

[17] Revels SL, Morris AM, Reddy RM, Akateh C, Wong SL (2013). Racial disparities in esophageal cancer outcomes. Ann Surg Oncol.

[18] Taioli E, Wolf AS, Camacho-Rivera M, Kaufman A, Lee DS, Bhora F, Flores RM (2016). Racial disparities in esophageal cancer survival after surgery. J Surg Oncol.

[19] Paulson EC, Ra J, Armstrong K, Wirtalla C, Spitz F, Kelz RR (2008). Underuse of esophagectomy as treatment for resectable esophageal cancer. Arch Surg.

[20] Polednak AP (2003). Trends in survival for both histologic types of esophageal cancer in US surveillance, epidemiology and end results areas. Int J Cancer.

[21] Stein HJ, Feith M, Bruecher BL, Naehrig J, Sarbia M, Siewert JR (2005). Early esophageal cancer: pattern of lymphatic spread and prognostic factors for long-term survival after surgical resection. Ann Surg.

[22] Siewert JR, Stein HJ, Feith M, Bruecher Bö LDM, Bartels H, Fink U (2001). Histologic tumor type is an independent prognostic parameter in esophageal cancer: lessons from more than 1,000 consecutive resections at a single center in the western world. Ann Surg.

[23] Brown LM, Hoover R, Silverman D (2001). Excess incidence of squamous cell esophageal cancer among US black men: role of social class and other risk factors. Am J Epidemiol.

[24] Brown LM, Hoover RN, Greenberg RS (1994). Are racial differences in squamous cell esophageal cancer explained by alcohol and tobacco use?. J Natl Cancer Inst.

[25] Ferraroni M, Negri E, La Vecchia C, D'Avanzo B, Franceschi S (1989). Socioeconomic indicators, tobacco and alcohol in the aetiology of digestive tract neoplasms. Int J Epidemiol.

[26] Pukkala E, Teppo L (1986). Socioeconomic status and education as risk determinants of gastrointestinal cancer. Prev Med.

[27] Zhang P, Wang J, Lu T (2015). miR-449b rs10061133 and miR-4293 rs12220909 polymorphisms are associated with decreased esophageal squamous cell carcinoma in a Chinese population. Tumour Biol.

[28] Iuliano R, Vismara MFM, Dattilo V, Trapasso F, Baudi F, Perrotti N (2013). The role of microRNAs in cancer susceptibility. Biomed Res Int.

[29] Ma WJ, Lv GD, Zheng ST (2010). DNA polymorphism and risk of esophageal squamous cell carcinoma in a population of North Xinjiang, China. World J Gastroenterol.

[30] Yang Y, Fa X (2015). Role of IL-10 gene polymorphisms on the susceptibility for esophageal cancer and its association with environmental factors. Int J Clin Exp Pathol.

